# Health Related Quality of Life in Individuals Transferred from a Needle Exchange Program and Starting Opioid Agonist Treatment

**DOI:** 10.1155/2018/3025683

**Published:** 2018-12-19

**Authors:** Martin Bråbäck, Louise Brådvik, Katja Troberg, Pernilla Isendahl, Suzan Nilsson, Anders Håkansson

**Affiliations:** ^1^Addiction Center Malmö, Division of Psychiatry, Sweden; ^2^Lund University, Faculty of Medicine, Department of Clinical Sciences Lund, Psychiatry, Lund, Sweden; ^3^Department of Infectious Diseases, University Hospital Skåne, Malmö, Sweden

## Abstract

**Background:**

Opioid agonist treatment (OAT), for the treatment of heroin dependence, has been reported to improve overall health and lower mortality. Drug use and retention in treatment have often been used as measures of treatment success. More recently, however, researchers have suggested that measurements of quality of life should be an outcome in substance use treatment evaluations. In a recent randomized controlled trial we demonstrated high rates of successful rapid referral from a needle exchange program (NEP) to OAT. The aim of this study was to see whether an improvement in health related quality of life (HRQoL) could be seen at 3-month follow-up after starting OAT and whether it was associated with any baseline characteristics. We also wanted to compare our sample to a sample from the general population with regard to HRQoL.

**Methods:**

This was a 3-month follow-up of 71 patients who started OAT. Measurements of HRQoL with EQ-5D (an instrument developed by the EuroQol group) were made at baseline and at three months.

**Results:**

Mean EQ-5D VAS (visual analogue scale) for the study sample at baseline was 47.3, which was lower than a Swedish reference population reporting 83.3. Individuals reporting being prescribed a drug for a psychiatric condition had significantly lower EQ-5D index values. Improvement in EQ-5D index score was significantly less for individuals reporting previous overdoses (-0.10, p=0.025). Individuals reporting previous suicide attempts had significantly lower EQ-5D VAS score at baseline. A significant increase of the EQ-5D VAS difference over time was found with a mean difference of 10.94 (p=0.008) for the total sample.

**Conclusion:**

To our knowledge this is the first time HRQoL as an outcome is reported in a population transferred from a NEP to OAT. Our results indicate that OAT can result in increased HRQoL, even with this type of rapid low-threshold referral.

## 1. Introduction

Heroin dependence is a chronic relapsing disorder with high mortality [1–3]. Opioid agonist treatment (OAT) with methadone or buprenorphine has been shown to increase social stability, improve somatic and psychiatric health, and lower overall mortality [4–6].

Traditionally when measuring treatment success we often look at the effect on drug use and retention in treatment. More recently, authors have called for a more holistic, consumer-oriented perspective on treatment success, since staff and patients have been found not always to have the same view on what could be called a successful treatment [7–9]. Therefore, as a complement to “hard” outcomes like retention in treatment and drug use, several researchers suggest that measurement of quality of life (QoL) as an outcome that should be part of any substance use treatment evaluation [10–13]. The field of substance abuse lags behind when it comes to these kinds of measurements; reporting of QoL is still rare and is also complicated by the fact that, when performed, several different instruments and methods are used, making comparisons difficult [13–15]. There are also differences between the two constructs QoL and health related quality of life (HRQoL). The latter addresses the individuals' self-reported health and ability to function in different domains concerning everyday life. HRQoL is focused on limitations caused by disease on physical, psychological, and social functioning [16], but it is sometimes misused as a synonym for QoL [17–19], which is often considered a broader concept including life domains beyond health, for example, living environment and satisfaction with life in general [13, 20]. Some authors advocate the use of QoL measurements in opioid-dependent people for a more holistic approach to treatment [13, 14, 21]. Others argue in favour of the use of HRQoL measures to be able to make calculations of quality-adjusted life years (QUALYs), which incorporates quantity of life in addition to HRQoL in a single value, as an outcome important for policy makers and when making cost-effectiveness analyses when it comes to treatment of opioid dependence. At the same time they report that the use of validated HRQoL instruments is still rare in published studies regarding opioid use disorder [15].

Studies have reported that opioid-dependent individuals seeking treatment report low HRQoL as compared to the general population [22–25]. They usually report poor scores for mental health but higher ones for physical functioning [14]. Still, the initiation and retention in OAT have been shown to improve HRQoL [23, 26–33]. Factors associated with lower HRQoL among opioid-dependent individuals have been higher age [34–36], female sex [22, 37, 38], psychiatric and physical comorbidity [28, 35, 39–45], and continued use of illicit substances [41]. Examples of factors associated with improved HRQoL among opioid-dependent individuals have been improved housing conditions [44, 46], decreases in illicit drug use [44], and social support [47].

In a previous study we reported an opioid-dependent cohort, recruited from a needle exchange program (NEP) in Malmö, Sweden. The included participants were characterized by a high degree of drug use severity, social problems, and physical and psychiatric comorbidity, but a majority could still be rapidly transferred to OAT [48]. In this paper we report measurements of HRQoL at baseline and three months into treatment using an instrument developed by the EuroQol group, EQ-5D [49], which has been validated for heroin-dependent patients [50] and shown to be responsive to decreases in illicit drug use [51]. To our knowledge, evaluating HRQoL as an outcome measure has never been done in a population rapidly transferred from a NEP to evidence-based treatment with buprenorphine/naloxone or methadone.

Aims of this study were (1) a comparison of the present sample with a reference sample from the general population regarding HRQoL, (2) a comparison of variables at baseline with index scores, and (3) an investigation of overall improvement in EQ-5D scores, and the possible association with any baseline variable or use of illicit drugs during treatment.

## 2. Materials and Methods

### 2.1. Setting

This study took place in Malmö which is a city with a population of roughly 300000 and situated in the southern part of Sweden. Maintenance treatment with methadone was introduced in Sweden in the late 60s and buprenorphine in 1999. Traditionally, Sweden has had high thresholds to maintenance treatment and the access has been limited in many areas. The treatment is only allowed at special addiction treatment units [52]. The NEP in Malmö was started in the 80s and is run by the Department for Infectious Diseases. It was not until 2006 that Sweden passed legislation that allowed needle exchange programs to be started also in other areas of Sweden. The legislation stated that besides preventing hepatitis and HIV needle exchange programs should motivate patients for treatment of drug dependence.

### 2.2. Participants and Procedures

The patient inclusion for the study took place between October 21, 2011, and April 1, 2013. The study was approved by the Regional Ethics Board Lund, Sweden. The institutional body supporting the study (the National Swedish Research Council for Working Life and Social Sciences) had no role in study design, data collection, data analysis, data interpretation, or writing of the report. All included patients signed informed consent. The patients were recruited to OAT at the NEP in Malmö. As we have reported in a previous study 71 out of 75 individuals successfully started maintenance treatment with buprenorphine or methadone at the OAT outpatient clinic [48]. Out of the four participants who did not start OAT one was arrested on her way to the OAT clinic and three did not show-up for start of OAT. The choice of medication for agonist treatment was made outside of the study protocol and the decision was based on individual clinical characteristics. Buprenorphine-naloxone was the first choice if the participant had not been in treatment before.

The individuals who entered treatment had a mean age of 39 ± 8.6; fifty-two were males and 19 females. The most common stated sources of income were social welfare (61%) and criminal activities (55%). Nine percent reported being employed and 31% reported that they had their own apartment. On average they reported injecting 21 of 30 days the last month prior to entering treatment and the mean age for starting using heroin was 21. Eighty percent reported having hepatitis C, 31 percent had previous suicide attempts, and 72 percent had experienced opiate overdoses. Polydrug use was common and roughly 73 percent reported using sedative-hypnotics the last 30 days prior to the baseline interview and 87 percent were positive for another illicit substance in the toxicology screen that was performed before initiating OAT.

Out of the 71 individuals who started OAT, 67 patients (94%) were still in treatment after three months [53]. In-treatment patients had to leave observed toxicology screens twice a week. After three months, they were designated responders in treatment if 80 percent of the samples were negative for opioids and/or other illicit drugs.

The assessment took place at the NEP at baseline and after three months in treatment at the OAT outpatient clinic.

### 2.3. Measures

EQ-5D is a generic instrument measuring HRQoL developed by the EuroQol group [54]. It assesses five different domains of health and functioning (mobility, self-care, usual activities, pain/discomfort, and anxiety/depression) with three severity levels. The scores result in 243 different health profiles. The Swedish experienced-based value set was used to determine EQ-5D index values for each health state [55]. The index value attached to each health state is on a scale between 1 (full health) and 0 (dead). The EQ-5D instrument includes a visual analogue scale (EQ-5D VAS) ranging from 0 to 100 where respondents rate their overall health status. The instrument can be used not only to measure the burden of disease but also to compare with samples from the general population [56].

### 2.4. Statistical Analysis

The difference of EQ-5D score at baseline and at three months was calculated and the variable was named “EQ-5D difference”. The EQ-5D VAS difference was also calculated in the same manner. A linear regression analysis was made with the “EQ-5D difference” and the “EQ-5D VAS difference” as dependent variables. Sex, age, randomization group, being responder in treatment (defined as more than 80 percent negative toxicology screens for opioids or other illicit substances for first three months in treatment), previous suicide attempts, and previous overdoses were analyzed as independent variables. Student's t-test was used in the analysis of whether there was a statistically significant increase of the “EQ-5D difference” or “EQ-5D VAS difference” over time. The Mann–Whitney test was used for group comparisons at baseline. The statistical analysis was made with IBM SPSS Statistics (version 22).

## 3. Results

**Figure 1:**
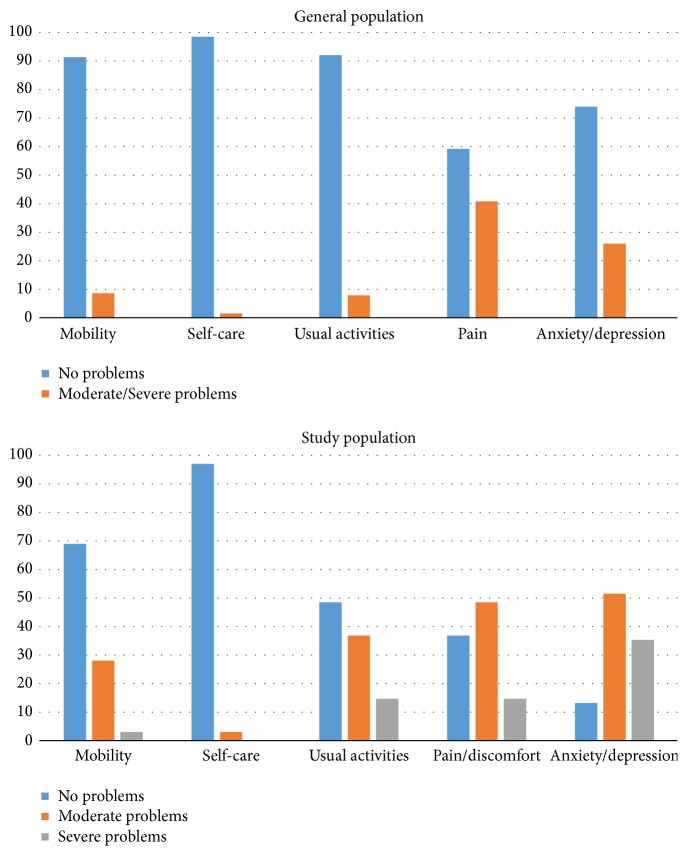
Percentage of individuals reporting problems in each EQ-5D domain in the study population compared to a general population sample from Sweden.

Mean EQ-5D VAS for the study sample at baseline was 47.3 which was considerably lower than a Swedish reference population who reported 83.3. More problems were also reported for all EQ-5D domains when compared to the general population sample with the highest percentage of reported problems from the domains of pain and anxiety/depression (Figure 1).

Individuals reporting being prescribed drugs for psychiatric conditions had significantly lower EQ-5D index values (Table 1). Individuals reporting previous suicide attempts had significantly lower EQ-5D VAS score at baseline. Individuals who were classified as responders (with 80 percent negative screening for other illicit drugs) had higher mean EQ-5D index score and EQ-5D VAS score.

When performing a linear regression analysis it was found that improvement in EQ-5D index score was significantly less for the individuals with previous overdoses (-0.10, p=0.025). A significant increase of EQ-5D score difference over time was not found. A significant increase of the EQ-5D VAS difference over time was found with a mean difference of 10.94 (p=0.008) for the whole group.

## 4. Discussion

To our knowledge, this is the first time HRQoL outcomes are reported in a population recruited from a NEP and rapidly referred to OAT. Mean EQ-5D VAS at baseline was found to be considerably lower than for a sample from the general population [56]. At baseline, mean EQ-5D VAS scores were significantly lower for individuals reporting previous suicide attempts as were mean EQ-5D index scores for individuals taking a psychiatric medication, possibly indicating that more psychological problems were correlated with decreased HRQoL at baseline. Having fewer problems with polydrug use at 3-month follow-up was significantly associated with higher EQ-5D index and EQ-5D VAS scores at baseline. Improvement in EQ-5D index score was significantly less for individuals reporting previous overdoses. A significant increase of the EQ-5D VAS difference was found over time for the whole sample indicating that treatment resulted in improved HRQoL.

Sun et al. reported mean EQ-5D VAS scores for a population of homeless individuals from the central part of Sweden [57]. They found the mean EQ-5D VAS score to be 62.6 for women and 54.9 for men; thus, it was lower compared to the general population but higher when compared to what we report for our sample (Table 1). When looking at the different domains at baseline, our population reported the most problems regarding anxiety/depression (89.8 percent), which was in accordance with Sun et al. describing homeless individuals to report the most problems in the same domain but in contrast to the reference population reporting the most problems from the domain of pain.

Most of the individuals included in the study were using other illicit substances, apart from opioids. High rates indicating polydrug use are usually common among this population of opioid-dependent individuals, in accordance with reports from other authors [58, 59]. Individuals, who at three months had 80 percent negative toxicology screens for other substances than opioids and hence were designated responders, had significantly higher mean EQ-5D index score and EQ-5D VAS score at baseline. Possibly, this may indicate that less extensive polydrug use problems are associated with higher HRQoL, as has been reported previously [25, 35].

Since psychiatric comorbidity is common in opioid dependence [60, 61] and as these problems have been found to lower HRQoL [35, 39, 40] our results were not surprising. At the same time OAT has been shown to improve psychological health already during the first month of treatment [62] and more recently it has been reported to improve HRQoL as well [26–33]. In line with those results, we report a significant increase of the EQ-5D VAS difference over time. However, we could not see any significant increase of the EQ-5D index score over time for the included individuals. For individuals reporting previous overdoses the difference in EQ-5D index score was, however, found to be significantly less.

There are some limitations to our study. On a more general level it is difficult to compare QoL and HRQoL between studies since the instruments measure different aspects of what we consider to represent QoL. We chose to use EQ-5D, since it has been previously validated to an opioid-dependent population. At the same time it is difficult to compare our results to studies using other HRQoL instruments. Another limitation is the Swedish reference population provided by the EuroQol group for comparison, as it was relatively small and due to the fact that the EQ-5D assessment was carried out more than twenty years ago. In addition the reference population was not matched with regard to sex and age when compared to the study population [63]. Another limitation is our limited sample size. However, we could, despite that, report some statistically significant results. Major strengths of the study were the fact that all included individuals were transferred to the same OAT outpatient clinic and assessed by the same physician and research personnel, making follow-up as consistent as possible.

## 5. Conclusions

We have previously presented a low-threshold procedure for rapidly referring opioid-dependent individuals from a NEP to evidence-based treatment with methadone and buprenorphine [48]. Despite having a high degree of problems due to use of illicit substances, signs of severe psychiatric symptomatology, and social problems, patients were retained in treatment to a high degree at 12-month follow-up [53]. In conclusion, authors have however suggested that patients and staff are not always in agreement with what characterizes efficient OAT [7], that retention and reduction in substance use and other “hard” data only describe part of the truth when it comes to treatment success, and that quality of life measures should be used as part of outcome evaluation [10–12]. The present study was a 3-month follow-up of individuals rapidly transferred from a NEP to OAT with regard to HRQoL. Our results indicate that OAT can result in an increase in HRQoL even with this way of low-threshold referral.

## Figures and Tables

**Table 1:** Mean EQ-5D index scores and EQ-5D VAS scores in relation to particular reported baseline characteristics for 71 individuals starting OAT.

		EQ-5D	p	EQ-5D VAS	p
	n (%)	mean (s.d)		mean (s.d)	
Male	53 (74.6)	0.73 (0.17)	0.926	48.9 (24.9)	0.469
Female	18 (25.4)	0.74 (0.13)		42.6 (28.1)	
Previous suicide attempts					
YES	22 (31.0)	0.69 (0.18)	0.193	36.5 (25.9)	0.022
NO	49 (69.0)	0.76 (0.15)		51.8 (24.5)	
Prescribed psychiatric medication					
YES	18 (25.4)	0.66 (0.16)	0.024	38.1 (22.5)	0.078
NO	53 (74.6)	0.76 (0.15)		50.4 (26.2)	
Using benzodiazepines last 30 days					
YES	52 (73.2)	0.72 (0.16)	0.055	44.2 (26.5)	0.086
NO	19 (26.8)	0.80 (0.12)		56.2 (21.5)	
Responders other illicit drugs					
YES	9 (13.2)	0.89 (0.10)	0.004	63.9 (21.7)	0.038
NO	59 (86.8)	0.72 (0.16)		45.6 (25.1)	
Responders opioids					
YES	21 (30.9)	0.80 (0.14)	0.129	55.8 (25.9)	0.118
NO	47 (69.1)	0.72 (0.16)		44.6 (24.7)	
Previous overdoses					
YES	51 (71.8)	0.74 (0.16)	0.940	44.4 (24.8)	0.182
NO	20 (28.2)	0.74 (0.15)		54.7 (27.2)	

## Data Availability

The data used to support the findings of this study are available from the corresponding author upon request.
